# Efficient Approach for the Extraction and Identification of Red Pigment from *Zanthoxylum bungeanum* Maxim and Its Antioxidant Activity

**DOI:** 10.3390/molecules23051109

**Published:** 2018-05-08

**Authors:** Xi Chen, Zhiqiang Wei, Lei Zhu, Xing Yuan, Daneng Wei, Wei Peng, Chunjie Wu

**Affiliations:** 1 College of Pharmacy, Chengdu University of Traditional Chinese Medicine, Chengdu 611137, China; cxcdutcm@163.com (X.C.); wzqcdutcm@163.com (Z.W.); zld163wyyx@163.com (L.Z.); yuanxing19891222@163.com (X.Y.); n403034985@163.com (D.W.); pengwei002@126.com (W.P.); 2 Key Research Laboratory of Traditional Chinese Medicine Processing Technology, State Administration of Traditional Chinese Medicine of China, Chengdu 611137, China

**Keywords:** *Zanthoxylum bungeanum* Maxim, red pigment, ultrasound-assisted extraction, BBD-RSM, UPLC-MS, antioxidant activity

## Abstract

Red pigment (RP) was extracted from the peels of *Zanthoxylum bungeanum* Maxim (PZB) by ultrasonic-assisted extraction (UAE) in this work. Box–Behnken design–response surface methodology (BBD-RSM) was employed to research the efficiency of the RP extraction. Based on the optimization of RSM, results showed that the optimal extraction conditions were as follows: liquid–solid ratio of 31 mL/g, extraction time of 41 min, and extraction temperature of 27 °C, and under these conditions, the actual absorbance value was 0.615 ± 0.13%, highly agreeing with the predicted value by the model. Furthermore, ultra-performance liquid chromatography–mass spectrometry (UPLC-MS) was used to separate and analyze RP. The compounds of RP were mainly flavonoids, and there were five compounds detected for the first time in PZB. In addition, RP showed significant antioxidant activities in vitro, which could be developed for anti-aging candidate drugs and functional foods. In conclusion, ultrasound-assisted extraction with BBD-RSM and chromatographic separation technology with UPLC-MS are efficient strategies for the isolation and identification of RP from PZB.

## 1. Introduction

*Zanthoxylum bungeanum* Maxim (Rutaceae), a known traditional spice in China, is commonly applied in cooking for its special flavor and numbing taste. *Z. bungeanum* has also been used as a common traditional Chinese medicine (TCM) for treating various diseases for decades. *Z. bungeanum* is widely distributed in China, including Sichuan, Shanxi, Gansu, Shandong, Hebei, etc. [1,2]. Increasing evidence has demonstrated that *Z. bungeanum* shows diversified pharmacological activities, such as insecticidal, antifungal, antioxidant, anti-hypertension, and anti-inflammatory effects, etc. [3,4,5,6,7]. In addition, previous investigations also reported that *Z. bungeanum* contains many compounds, including alkaloids, terpenoids, flavonoids, free fatty acids, etc. [8]. Furthermore, amides (the characteristic alkaloids in this plant), essential oil, and flavonoids are reported to be the major active constituents of *Z. bungeanum* [4,9].

Color, aroma, and numbing taste are the most important indices of the commercial quality of the peels of *Z. bungeanum* (PZB). Color is the most visualized evaluation indicator which largely impacts the purchase behavior of consumers, and red pigment (RP) is the material basis of PZB’s color. In recent years, synthetic colorants abuse has gradually attracted public attention. In view of the harm caused by synthetic colorants added in food, an increasing number of people tend to purchase natural pigments which are allergen-free, biodegradable, nontoxic, nonpolluting, and non-carcinogenic [10,11]. The RP from PZB could be a novel natural colorant added to food. Investigations have revealed that natural pigments possess various biological effects, including antioxidant activity, antimicrobial effect, immune stimulation, and improving lipid metabolism [12,13,14,15].

Conventional solvent extraction techniques for pigments such as maceration are time-consuming and high consumption of extraction solvent [10]. The bioactivity of pigments is susceptible to extraction conditions such as long extraction time and high temperature; therefore, a suitable, innovative, and effective extraction strategy appears to be urgently needed. Compared with conventional solvent extraction techniques, ultrasound-assisted extraction (UAE) has the advantages of high efficiency, short time, low cost, and high automatization based on its mechanical effect and acoustic cavitation [16]. UAE is commonly used in pigment extraction, as it can prevent the active ingredients of natural pigment from structural damage and maintain the biological activity as much as possible [17,18,19].

When using UAE for the extraction of active ingredients, there are many extraction conditions to optimize, including liquid–solid ratio, extraction time, extraction temperature, ultrasonic power, ultrasonic frequency, etc. These variables interact with each other and have a joint impact on the desired response. In this case, response surface methodology (RSM), combining statistical and mathematical systems successfully, is more effective, time-saving, and convenient to optimize the process than other classical methods [20,21]. The Box–Behnken design (BBD) an RSM design which can be used to establish a second-order polynomial model to obtain the optimum extraction parameters [22]. Furthermore, a single-factor experiment should be performed to make the variables and factors simplified before the RSM [23].

Ultra-performance liquid chromatography (UPLC), a superior form of liquid chromatography (LC), has advantages in analysis speed and separation capacity [24]. The UPLC separation column is packed with smaller particles (<2 µm) and can support high pressures in operating systems. Combining UPLC with mass spectrometry (MS) has the features of high efficiency, high sensitivity, and low consumption. UPLC-MS has played a significant role in the effective separation and structural analysis of chemical components [25].

To the best of our knowledge, there are few studies on RP from PZB. A Chinese scholar has studied the extraction process of pigment from PZB with an orthogonal design method by maceration, and speculated that the pigment belonged to anthocyanins [26]. However, the extraction with an orthogonal design method is not sufficient to optimize the extraction conditions, and no studies have yet reported the chromatographic separation and identification of RP from PZB and its antioxidant activity. Therefore, it is necessary to research the extraction, separation, and identification of RP from PZB.

UAE was applied in this study, and the extraction conditions were optimized by the Box–Behnken design–response surface method (BBD-RSM) to acquire a more efficient method for the extraction of RP from PZB. Furthermore, UPLC-MS was performed to separate and identify the RP from PZB. Furthermore, free radical scavenging assays were performed to investigate the in vitro antioxidant activity of RP from PZB.

## 2. Results and Discussion

### 2.1. Spectral Characteristics of RP

**Figure 1:**
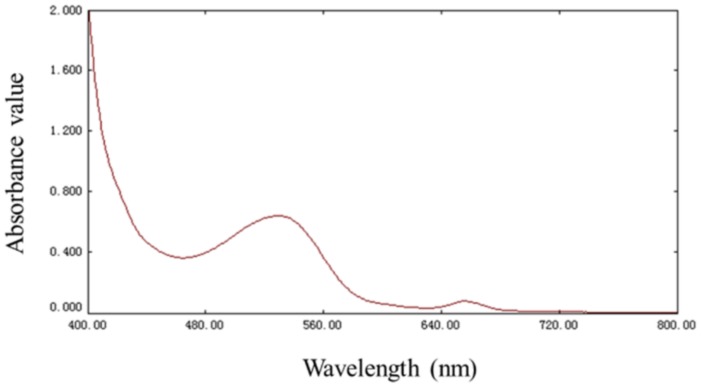
Spectral characteristic of red pigment (RP) in the wavelength range of 400 to 800 nm.

As displayed in Figure 1, the RP of PZB had an obvious absorption at 520 nm, which is the characteristic absorption wavelength of anthocyanins [27]. Because 520 nm was the maximum absorption wavelength of RP, the absorbance value of RP at 520 nm manifested the extraction efficiency of RP and was used as the response value for the experimental design.

### 2.2 Single-Factor Experiment Analysis

#### 2.2.1. Effects of Solvents on RP Extraction

The effects of solvents on RP extraction are shown in Table 1. In this study, the extraction efficiency of different extraction solvents was investigated with the other factors set at the central point. It was found that the extraction efficiency of 0.1% (*v*/*v*) hydrochloric acid in methanol was the best according to the color and absorbance value of RP. Further, it is advantageous to extract anthocyanin pigments in acidic solvents such as hydrochloric acid, as it can keep the pigment stable and reduce its degradation [28]. Hence, 0.1% (*v*/*v*) hydrochloric acid in methanol was chosen as the optimum extraction solvent of RP and used for further experiments.

#### 2.2.2. Effects of Liquid–Solid Ratio on RP Extraction

**Figure 2:**
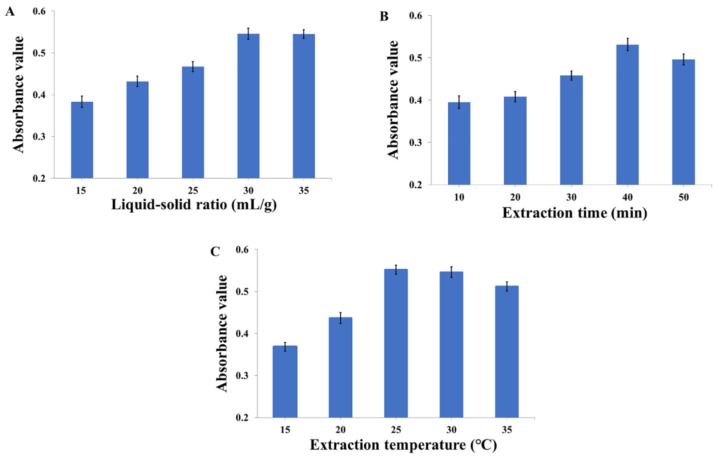
Effects of (**A**) liquid–solid ratio, (**B**) extraction time, and (**C**) extraction temperature on the absorbance value of RP.

The effects of liquid–solid ratio (15, 20, 25, 30, 35 mL/g) on the extraction efficiency of RP were investigated, and the results are displayed in Figure 2A. Meanwhile, other parameters were fixed as follows: the extraction solvent was 0.1% (*v*/*v*) hydrochloric acid in methanol, the extraction time 30 min, and temperature 25 °C. The absorbance value of RP obviously increased before the liquid–solid ratio reached 30 mL/g, but after that, the absorbance value of RP changed slightly. The results showed that high liquid–solid ratios would lead to the waste of extraction solvent instead of improving the extraction efficiency significantly. Based on our results above, the liquid–solid ratios of 25–35 mL/g were selected for further experiments.

#### 2.2.3. Effects of Extraction Time on RP Extraction

The extraction times of 10, 20, 30, 40, and 50 min were studied to analyze the influence of extraction time on the extraction efficiency of RP while the other parameters were kept constant (the extraction solvent was 0.1% (*v*/*v*) hydrochloric acid in methanol, liquid–solid ratio 30 mL/g, and extraction temperature 25 °C), and the results are reported in Figure 2B. With the increase of extraction time, the absorbance value of RP increased at first and decreased afterwards, reaching a maximum at 40 min. This finding indicated that the extraction efficiency began to keep a dynamic equilibrium with the increase of extraction time. However, overlong extraction time would cause the degradation of RP [29]. Consequently, an extraction time of 30–50 min was used in the RSM experiments.

#### 2.2.4. Effect of Extraction Temperature on RP Extraction

The effects of extraction temperatures 15, 20, 25, 30, and 35 °C are reported in Figure 2C for the liquid–solid ratio of 30 mL/g and the extraction time of 30 min. The extraction efficiency of RP increased significantly when temperature changed from 15 °C to 25 °C, and reached a maximum level at 25 °C. This phenomenon indicated that a higher temperature initially increased the dissolution of RP, but when the temperature continued to rise, some heat labile components in RP were destroyed. Usually, the extraction temperature of anthocyanins should be controlled under approximately 30 °C [30]. Therefore, 20–30 °C was considered to be the extraction temperature range of the RSM experiments.

### 2.3. Optimization of Extraction Conditions

#### 2.3.1. Model Fitting Analysis

On the basis of the previous single-factor experiments, liquid–solid ratio (X_1_), extraction time (X_2_), and extraction temperature (X_3_) were chosen as the three factors of the response surface methodology (the extraction solvent was 0.1% (*v*/*v*) hydrochloric acid in methanol). As illustrated in Table 2, seventeen experiments were designed with BBD. The actual absorbance value of RP varied from 0.460 to 0.620, reaching a maximum value at a liquid–solid ratio of 30 mL/g, extraction time of 40 min, and extraction temperature of 25 °C. The predicted absorbance value of RP was gained from the second-order polynomial Equation (1):

A = 0.61 + 0.027·X_1_ + 0.011·X_2_ + 0.026·X_3_ + 0.013·X_1_X_2_ − 5.750 × 10^−3^·X_1_X_3_ − 4.250 × 10^−3^·X_2_X_3_ − 0.055·X_1_^2^ − 0.064 X_2_^2^ − 0.025·X_3_^2^. (1)

According to the results in Table 2, the analysis of variance (ANOVA) conducted to analyze the suitability and adequacy of the model is shown in Table 3. The regression models were tremendously significant from the perspective of high *F*-value (33.97) and low *p*-value (<0.0001). The lack of fit (*F*-value = 1.70, *p*-value = 0.3041 > 0.05) was not significant and indicated that the model could be applied to predict the absorbance value of RP. Both determination coefficient (R^2^ = 0.9776) and adjusted determination coefficient (Adj R^2^ = 0.9488) were significant and high, which showed that the predicted values were correlated with the experimental values. The low coefficient of variation (C.V.) (2.33%) and high Adeq precision (15.210) indicated that there was relatively high precision and reliability among the experimental values. In addition, the significance of each coefficient can be evaluated from the *F*-value (positively correlated with significance) and *p*-values (negatively correlated with significance). When the *p*-value of the model was less than 0.05, the model was significant and suited to optimize the extraction parameters. The independent variables (X_1_, X_2_, and X_3_) and the quadratic terms (X_1_^2^, X_2_^2^, and X_3_^2^) were significant. However, the other coefficients (X_1_X_2_, X_1_X_3_, X_2_X_3_) were not significant.

#### 2.3.2. Response Surface Analysis 

**Figure 3:**
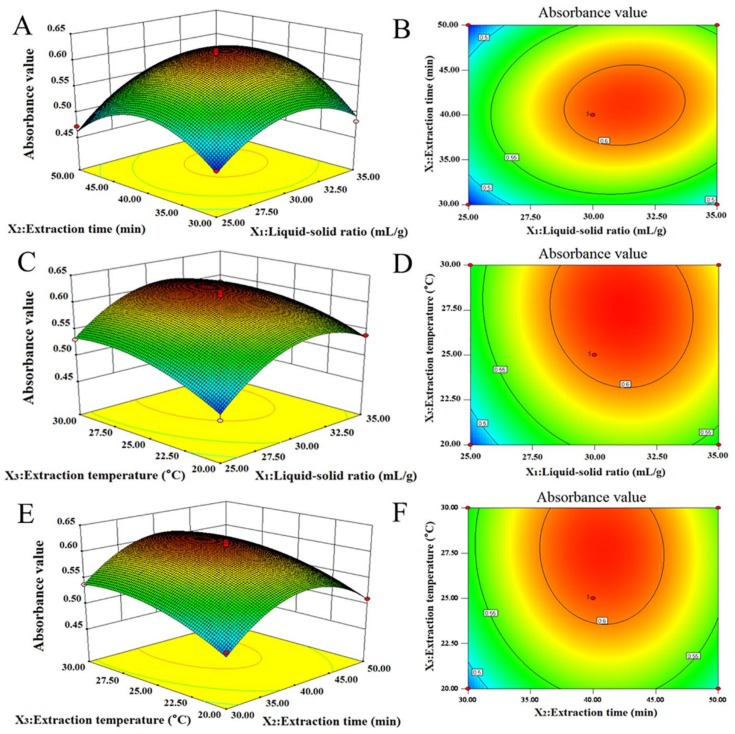
The 3D response surface and 2D contour plots showing the effects of liquid–solid ratio (mL/g), extraction time (min), and extraction temperature (°C) on the extraction efficiency of RP. (**A**,**B**) 3D response surface and 2D contour plots showing the effects of liquid–solid ratio and extraction time on the extraction efficiency of RP; (**C**,**D**) 3D response surface and 2D contour plots showing the effects of liquid–solid ratio and extraction temperature on the extraction efficiency of RP; (**E**,**F**) 3D response surface and 2D contour plots showing the effects of extraction time and extraction temperature on the extraction efficiency of RP.

As shown in Figure 3, a 3D response surface and 2D contour plots were used to analyze the interaction effects of three variables on the absorbance values of the RP on the basis of the regression equation. When the shape of contour plot is elliptic instead of round, the interaction between variables is significant. Otherwise, the interaction is insignificant. Besides, when the shape of the response surface is convex, the range of variables was set reasonably [31].

The effects of liquid–solid ratio, extraction time, and their interaction on the absorbance value of RP are revealed in Figure 3A,B. The RP absorbance value increased when the liquid–solid ratio increased in the range of 25–31.35 mL/g, and the extraction time increased in the range of 30–41.05 min, and then the absorbance value declined after 31.35 mL/g and 41.05 min. The circular contour shapes showed that the interaction of the liquid–solid ratio and extraction time was insignificant, which agrees with the results in Table 3. The effects of liquid–solid ratio and extraction temperature on the absorbance value of RP are shown in Figure 3C,D. It can be found that when the liquid–solid ratio was 30.99 mL/g and extraction temperature was 27.71 °C, the absorbance value of RP reached a maximum (approximately 0.618). The shape of the contour plot was circular, which indicates that the interactions of the two variables were not significant. As reported in Figure 3E,F, the effects of extraction time and extraction temperature on the absorbance of RP were evaluated. A maximum absorbance value of RP was obtained when the extraction time and extraction temperature was approximately 40.68 min and 27.85 °C. Additionally, the circular contour plots showed that the interaction between extraction time and extraction temperature was insignificant.

#### 2.3.3. Verification of Extraction Conditions

The feasibility of the model for calculating the optimum response values were tested with the optimum conditions as follows: the liquid–solid ratio was 31.14 mL/g, extraction time was 40.93 min, and the extraction temperature was 27.44 °C. Validation tests (*n* = 3) were executed under the aforesaid conditions with slight modifications: the liquid–solid ratio was 31 mL/g, the extraction time was 41 min, and the extraction temperature was 27 °C. Under these conditions, the actual absorbance of RP obtained was 0.615 ± 0.13%, highly matching with the predicted value and consequently indicating that the RSM model is accurate and adequate.

### 2.4. UPLC-MS Analysis

**Figure 4:**
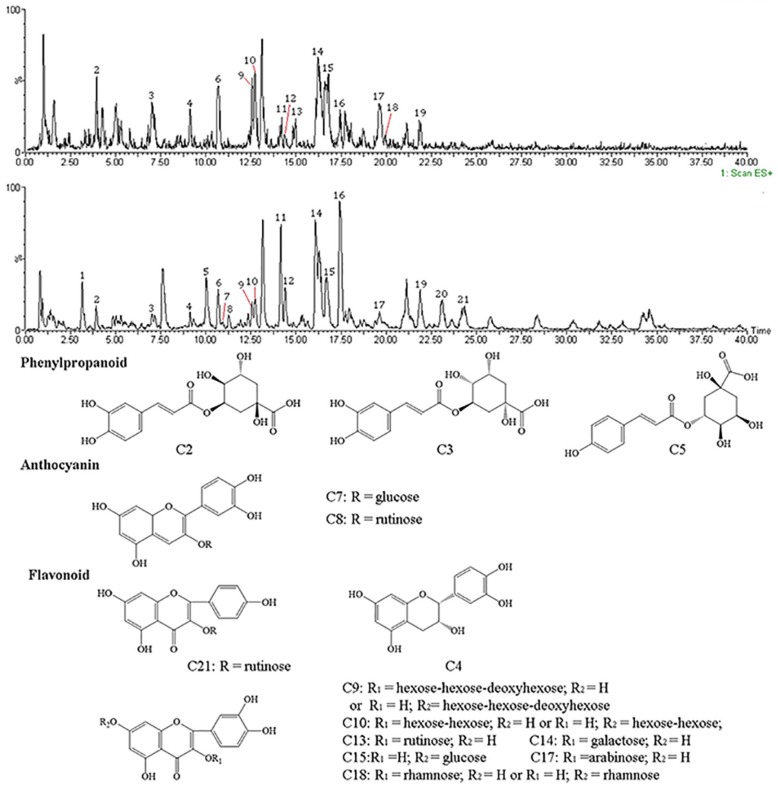
Total ion chromatogram and chemical structures of RP by ultra-performance liquid chromatography–mass spectrometry (UPLC-MS).

Chemical constituents of the RP from PZB were separated and identified using UPLC-MS at negative and positive modes, respectively, and the total ion chromatograms are presented in Figure 4. From our results, twenty-one constituents were detected, including phenylpropanoids, anthocyanins, and flavonoids. The related information is reported in Table 4, and the chemical structure is shown in Figure 4. Among them, five constituents (**2**, **5**, **7**, **8**, **18**) were identified for the first time in PZB. The compounds analysis was established on the MS, MS_2_ spectral data and the mass spectrometric data interpretation reported in the literature.

As shown in Table 4, compounds **2**, **3**, and **5** all belonged to the phenylpropanoid family. The MS data revealed that compounds **2** and **3** were isomers. Compound **2** with ions at *m*/*z* 353.36 [M − H]^−^ was identified as neochlorogenic, which have the fragment ion at *m*/*z* 191 [M − H − caffeoyl]^−^, *m*/*z* 179 [caffeic acid-H]^−^, *m*/*z* 173 [M − H − caffeoyl − H_2_O]^−^, *m*/*z* 161 [caffeic acid − H − H_2_O]^−^, *m*/*z* 135 [caffeic acid-H-CO_2_]^−^ [32]. According to the above-mentioned the fragmentation rules, compound **3** with ion at *m*/*z* 355.17 [M + H]^+^ was proposed as chlorogenic acid which yielded a product ion at *m*/*z* 175 [M + H − caffeoyl − H_2_O]^+^ and *m*/*z* 163 [caffeic acid + H − H_2_O]^+^. Compound **5** showed a molecular ion at *m*/*z* 337.24, a significant fragment ion at *m*/*z* 163 and an unobvious product ion at *m*/*z* 191, corresponding to *p*-coumaroylquinic acid on the basis of mass spectrometric data reported by Zhang et al. [33].

Compound **4** presenting a molecular ion at *m*/*z* 291.16 [M + H]^+^ was identified as epicatechin. The fragment ion at *m*/*z* 273 [M + H − 18]^+^ corresponds to the loss of H_2_O, *m*/*z* 165 [M + H − 126]^+^ corresponds to the elimination of phloroglucinol, which was consistent with the literature reported by Ivanova et al. [34]. Epicatechin belongs to catechins mainly sourced from tea polyphenols which has various bioactivities, such as antioxidant, anti-inflammatory, and antidiabetic effects, and so on [35].

There were two anthocyanins detected in the experiment. Compound **7** displaying an ion at *m*/*z* 449.14 [M]^+^ was characterized as cyanidin-3-glucoside. The product ion at *m*/*z* 287 [M − 162]^+^, generating from the elimination of a glucoside, matched with the cyanidin. Compound **8** showed a molecular ion at *m*/*z* 595.28 [M]^+^ and two fragment ions at *m*/*z* 449 [M − 146]^+^ (loss of rhamnose) and *m*/*z* 287 [M − 146 − 162]^+^ (loss of rutinose); it was analyzed as cyanidin-3-rutinoside [36].

Seven quercetin glycosides were identified as displayed in Table 4. Compound **9** and compound **10** manifested molecular ions at *m*/*z* 773.33 and *m*/*z* 627.25, both of which had obvious product ions at *m*/*z* 303 and matched with quercetin, revealing that compounds **9** and **10** were quercetin glycosides. However, there was no more fragmentation information to confirm sugars existing in the above quercetin glycosides. According to the displayed fragment information, compound **9** might be quercitrin triglycoside, and compound **10** was likely to be quercitrin bioside. The former lost 470 µ, possibly corresponding to two hexoses and one deoxyhexose. The latter lost 324 µ, probably conforming to two hexoses. The precursor ions at *m*/*z* 609.34, 463.28, 463.33, and 433.25 all generated product ions at *m*/*z* 300, which corresponds to a quercetin moiety. Combined with the reported components of PZB in the literature, compound **13** losing rutinose was identified as rutin. Compound **14** eliminating galactose was analyzed as hyperoside. Compound **15** was characterized as quercetin-7-glucoside by the loss of glucoside. Compound **17** was regarded as guaijaverin which lost arabopyranose. Compound **18** presenting a molecular ion at *m*/*z* 447 and a significant MS_2_ fragment ion at *m*/*z* 301 [M − H − 146]^−^ (loss of rhamnose) resulted as a quercetin moiety and a sugar moiety. Based on other small fragments at *m*/*z* 271, 255, 179, and 151, the compound was identified as quercetin-3-rhamnoside [37].

Compound **21** with a pseudo molecular ion at *m*/*z* 595.27 [M + H]^+^ presented an obvious fragment ion at *m*/*z* 287, resulting from the loss of rutinose. According to the reported compounds in *Z. bungeanum*, it was analyzed as kaempferol-3-rutinoside.

Seven compounds (**1**, **6**, **11**, **12**, **16**, **19**, and **20**) have not yet been identified. There were no mass spectral data in the databases or reported literature to characterize the above compounds. Further mass spectrometry analysis and NMR identification might be employed to determine these compounds.

### 2.5. Antioxidant Activity of RP

#### 2.5.1. 1,1-Diphenyl-2-picrylhydrazyl (DPPH)· Radical Scavenging Capacity of RP 

**Figure 5:**
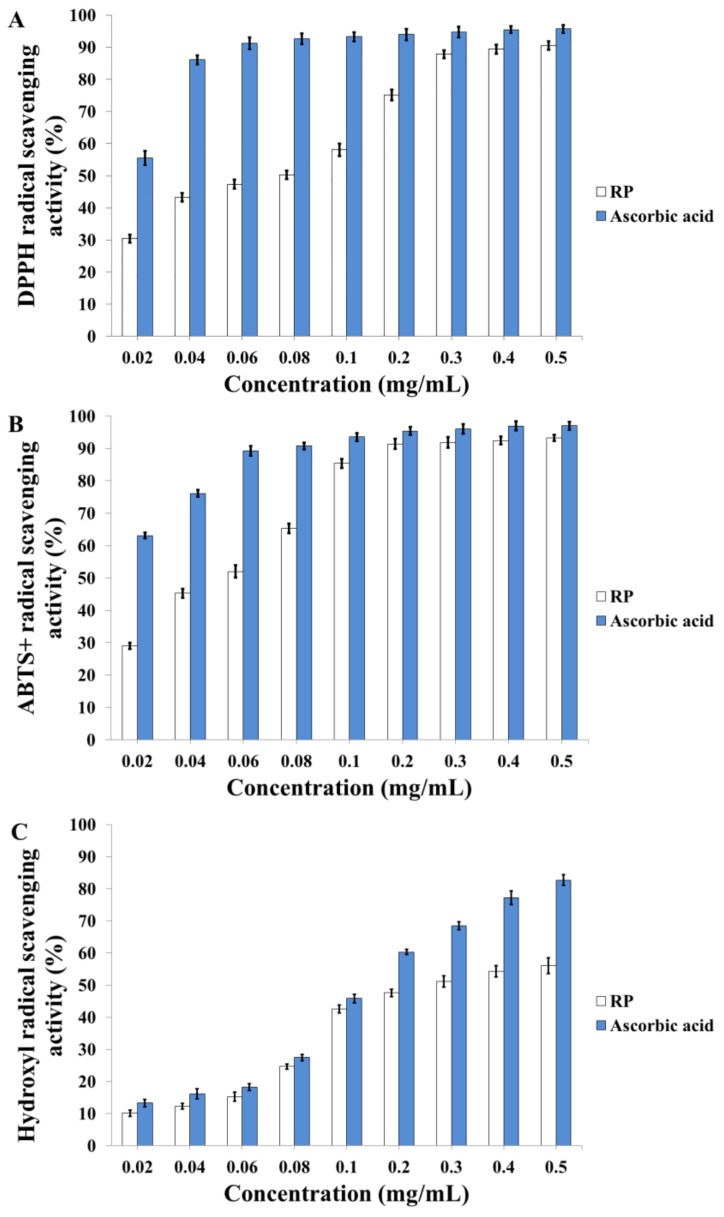
Antioxidant activities of RP. (**A**) 1,1-Diphenyl-2-picrylhydrazyl (DPPH)· radical scavenging assay; (**B**) 2,2'-Azinobis-(3-ethylbenzthiazoline-6-sulphonate) (ABTS)·^+^ radical scavenging assay; (**C**) Hydroxyl radical scavenging assay.

The DPPH· radical scavenging capacity assay is widely applied to evaluating the antioxidant activity of candidate agents, and is relatively convenient and rapid compared with other methods [38]. As Figure 5A indicates, in the concentration range of 0.02–0.5 mg/mL, the DPPH· scavenging rate of RP ranged from 30.45% to 90.50%, and the EC_50_ (efficient concentration to scavenge 50% radicals) of RP was 60 μg/mL. Therefore, our results revealed that RP has a potential scavenging capacity for DPPH· radicals.

#### 2.5.2. 2,2'-Azinobis-(3-ethylbenzthiazoline-6-sulphonate)(ABTS)·^+^ Radical Scavenging Capacity of RP

The ABTS·^+^ radical scavenging assay is another common method used to evaluate the antioxidant activity of chemical components [39]. As shown in Figure 5B, the ABTS·^+^ radical scavenging capacity of RP varied from 29.04% to 93.20% at the concentration of 0.02–0.5 mg/mL with an EC_50_ value of 42 μg/mL. The results showed that RP had a prominent ABTS·^+^ radical scavenging potential.

#### 2.5.3. Hydroxyl Radical Scavenging Capacity of RP

The hydroxyl radical scavenging assay is also adopted frequently for antioxidant activity evaluation [40]. As shown in Figure 5C, the hydroxyl radical scavenging capacity of RP ranged from 10.16% to 56.11% at the concentration of 0.02–0.5 mg/mL with an EC_50_ value of 268 μg/mL. The results indicated that RP had hydroxyl radical scavenging potential. However, the hydroxyl radical scavenging capacity of RP was poorer than the radical scavenging capacity of DPPH· and ABTS·^+^.

Aging is a complicated physiological process, and usually causes cognitive dysfunctions, memory loss, schizophrenia, Alzheimer’s and Parkinson’s diseases, etc. The biological mechanisms for explaining the aging process are not clear yet, and the free radical theory is one of the most approved theories [41]. Hence, antioxidant substances may be advantageous for delaying aging and extending the life span. Collectively, the results discussed above suggest that RP could be beneficial for scavenging body radicals, which could be further developed for anti-aging candidate drugs and foods.

## 3. Materials and Methods

### 3.1. Materials and Reagents

PZB was purchased from Hanyuan, Sichuan province, China, and authenticated by Professor Chunjie Wu (College of Pharmacy, Chengdu University of TCM, Chengdu, China). A specimen was stored at our laboratory (College of Pharmacy, Chengdu University of TCM, Chengdu, China). 1,1-Diphenyl-2-picrylhydrazyl (DPPH), 2,2'-azinobis-(3-ethylbenzthiazoline-6-sulphonate) (ABTS), and ascorbic acid were purchased from Sigma Chemicals Co. (St. Louis, MO, USA). All chemicals were of analytical grade, and the water used was purified water.

### 3.2. Ultrasound-Assisted Extraction (UAE)

In this study, PZB was pretreated with ethyl acetate to remove impurities and then air-dried. PZBs (2.0 g) were put into an Erlenmeyer flask with some extraction solution. Then, the Erlenmeyer flask with PZB and extraction solution was put in an ultrasonic extraction machine (Tianjin Autoscience Instrument Co., Ltd., Tianjin, China) with a power of 300 W and a frequency of 40 kHz and extracted at an appropriate extraction time and temperature. After extraction, the extract mixture was separated by filtering with a funnel, and the solution concentrated to 50 mL by rotary evaporator (Yarong Biochemical Instrument, Shanghai, China) under reduced pressure at 45 °C. Thus, the original pigment extraction for absorbance detection was obtained. In addition, the original pigment extraction was concentrated further to remove solvents by rotary evaporator, and an appropriate volume of ethyl acetate was put into the concentrates to get rid of amides, essential oil, and other compositions further. Then, primary purified RP was collected for the subsequent determination of antioxidant activity and identification.

### 3.3. Spectral Characteristics Analysis

To obtain the maximum absorption wavelength of RP, the spectral characteristics of the above original pigment extraction was detected by UV-Vis spectrophotometer (Purkinje General Instrument Co., Ltd., Beijing, China) in the range of 400 to 800 nm. Then, the absorbance of the original pigment extraction was determined at the maximum absorption wavelength to evaluate the extraction efficiency of RP according to the method previously reported by Liu et al. with some modifications [42].

### 3.4. Single-Factor Experiment

Single-factor experiments of extraction solvents, liquid–solid ratio, extraction time, and extraction temperature were carried out to simplify variables and factors. The conditions of extraction employed were as follows: different extraction solvents including ethanol, purified water, 60% ethanol, methanol, 0.1% (*v*/*v*) hydrochloric acid in ethanol, 0.1% (*v*/*v*) hydrochloric acid, 0.1% (*v*/*v*) hydrochloric acid in 60% ethanol, and 0.1% (*v*/*v*) hydrochloric acid in methanol were considered; liquid–solid ratios 15, 20, 25, 30, and 35 mL/g; extraction times 10, 20, 30, 40, and 50 min; and ultrasonic temperature 15, 20, 25, 30, and 35 °C.

### 3.5. Optimization of Extraction Technology by BBD-RSM

Based on the preliminary single-factor experiments, the liquid–solid ratio, extraction time, and extraction temperature were selected to be the three factors of the RSM design with the optimal solvents (0.1% (*v*/*v*) hydrochloric acid in methanol). A Box–Behnken design (BBD) of three factors at three levels was set to perform RSM by using Design Expert software (version 8.0.6.1, Stat-Ease Inc., Minneapolis, MN, USA). Three major influence factors covered liquid–solid ratio (X_1_, mL/g), extraction time (X_2_, min), and extraction temperature (X_3_, °C), and three levels coded −1, 0, and +1 were respectively built based on these factors (Table 5).

### 3.6. Separation and Identification of RP by UPLC-MS

In the research, UPLC-MS was applied to the identification of RP. The analysis was operated on an Acquity UPLC-Quattro Premier XE system (Waters, Massachusetts, USA). The sample separation was performed applying a UPLC HSS C18 analytical column (2.1 mm × 100 mm, 1.7 µm) (Waters, Massachusetts, USA) at 45 °C, the flow rate was 0.25 mL/min, and the injection volume was 2 µL. The mobile phase included methanol (solvent A) and 0.1% (*v*/*v*) formic acid in water (solvent B). The elution gradient started with 10–35% A to 15 min, 35–45% A to 40 min (end analysis). Mass spectra parameters were set as follows: negative and positive ion mode for a mass range of *m*/*z* 180 to *m*/*z* 800, capillary voltage 2.8 kV, cone voltage 20 V, source temperature 100 °C, desolvation temperature 250 °C, cone gas flow 40 L/h, desolvation gas flow 500 L/h.

### 3.7. Antioxidant Activity Assays of RP

#### 3.7.1. DPPH· Radical Scavenging Assay

The DPPH· radical scavenging activity was analyzed according to a method reported with some amendments [43]. A series of concentrations of RP (0.02–0.5 mg/mL) were prepared, and DPPH· solution (1 × 10^−4^ mol/L, 2 mL, in ethanol) was mixed with the sample solutions (2 mL), respectively. The mixture was kept in the dark at room temperature for 30 min, the absorbance was then measured at 517 nm, and ascorbic acid served as the positive control. The DPPH· free radical scavenging capability was calculated as the following Equation (2):

(2) $$\mathrm{DPPH}\cdot \text{free radical scavenging capability }(\%)\;=\;(1-\frac{A_{1}-A_{2}}{A_{0}})\times 100$$

where *A*_0_ is the absorbance of methanol plus DPPH· solution. *A*_1_ is the absorbance of samples plus DPPH·. *A*_2_ is the absorbance of samples plus ethanol.

#### 3.7.2. ABTS·^+^ Radical Scavenging Activity

The ABTS·^+^ radical scavenging capacity was evaluated on the basis of a previous report with some modifications [44]. A series of concentrations of RP (0.02–0.5 mg/mL) were prepared, ABTS·^+^ was generated after the chemical reaction between potassium persulphate solution (2.5 mM) and ABTS solution (7 mM) in the dark for 12–16 h at room temperature. The prepared ABTS·^+^ solution was diluted with purified water to the absorbance of 0.70 ± 0.02 at 734 nm. Then, the diluted ABTS·^+^ solution (3.5 mL) was added into sample solution (0.5 mL) and interacted for 5 min at room temperature. The absorbance was measured at 734 nm and the ascorbic acid served as a positive control. The ABTS·^+^ radical scavenging capacity was evaluated according to Equation (3):

(3) $${\mathrm{ABTS}}^{+}\text{free radical scavenging capability}(\%)\;=\;(1-\frac{A_{1}-A_{2}}{A_{0}})\times 100$$

where *A*_0_ is the absorbance of methanol plus ABTS·^+^ solution. *A*_1_ is the absorbance of samples plus ABTS·^+^. *A*_2_ is the absorbance of samples plus purified water.

#### 3.7.3. Hydroxyl Radical Scavenging Activity

The hydroxyl radical scavenging activity was evaluated based on the report described previously with some amendments [45]. Hydroxyl radicals, generated from the chemical reaction between FeSO_4_ and H_2_O_2_, have the ability to hydroxylate salicylate and the absorbance can be measured at 510 nm. A series of concentrations of RP (0.02–0.5 mg/mL) were prepared. RP solution (1 mL) was mixed with FeSO_4_ solution (6 mM, 1 mL), H_2_O_2_ solution (6 mM, 1 mL), and salicylic acid solution (6 mM, 1 mL, in ethanol), and reacted at 37 °C for 30 min. The absorbance was measured at 510 nm and ascorbic acid was used as the positive control. The hydroxyl radical scavenging activity was calculated as follows:

(4) $$\text{Hydroxyl free radical scavenging capability}(\%)\;=\;(1-\frac{A_{1}-A_{2}}{A_{0}})\times 100$$

where *A*_0_ is the absorbance of methanol plus FeSO_4_, H_2_O_2_, and salicylic acid solution. *A*_1_ is the absorbance of samples plus FeSO_4_, H_2_O_2_, and salicylic acid solution. *A*_2_ is the absorbance of samples plus FeSO_4_, H_2_O_2_, and ethanol.

### 3.8. Statistical Analysis

Three parallel tests were carried out in each experiment, and results were represented as means ± S.D. Analysis of variance (ANOVA) was utilized to analyze the data, and *p* < 0.05 was deemed statistically significant.

## 4. Conclusions

In this research, the RP from PZB was extracted efficiently by utilizing an ultrasound-assisted method in combination with BBD-RSM. The optimal conditions were as follows: liquid–solid ratio of 31 mL/g, extraction time of 41 min, and extraction temperature of 27 °C. The actual absorbance value was 0.615 ± 0.13%, highly agreeing with the value predicted by the model.

In addition, the chromatographic separation and characterization of the RP from PZB were investigated tentatively for the first time in the present study. The RP from PZB was separated and identified by UPLC-MS. The results indicated that compounds of RP from PZB including phenylpropanoids, anthocyanins and flavonoids were detected, and quercetin glycosides were the major compounds. Neochlorogenic acid, *p*-coumaroylquinic acid, cyanidin-3-glucoside, cyanidin-3-rutinoside, and quercetin-3-rhamnoside were detected from PZB for the first time.

Furthermore, the in vitro antioxidant activity suggested that the RP of PZB has significant free radical scavenging capacity and could be developed for anti-aging drugs and functional foods. In conclusion, this research provides scientific data for the highly-efficient extraction and identification of RP from PZB.

## Figures and Tables

**Table 1:** Effects of different extraction solvents on the absorbance value of RP.

Extraction Solvents	Color	Absorbance Value
ethanol	pale yellow	0.080
purified water	pale yellow	0.110
60% ethanol	yellow	0.140
methanol	yellow	0.160
0.1% (*v*/*v*) hydrochloric acid in ethanol	pale red	0.253
0.1% (*v*/*v*) hydrochloric acid	pale red	0.291
0.1% (*v*/*v*) hydrochloric acid in 60% ethanol	red	0.521
0.1% (*v*/*v*) hydrochloric acid in methanol	red	0.614

**Table 2:** Experimental design and absorbance values with Box–Behnken design.

Run	Liquid–Solid Ratio X_1_ (mL/g)	Time X_2_ (min)	Temperature X_3_ (°C)	Absorbance Value	
				Actual Value	Predicted Value
1	0	0	0	0.613	0.610
2	0	0	0	0.598	0.610
3	−1	0	−1	0.460	0.470
4	1	0	−1	0.539	0.540
5	0	1	−1	0.510	0.510
6	0	0	0	0.617	0.610
7	−1	0	1	0.531	0.530
8	1	1	0	0.536	0.540
9	0	0	0	0.620	0.610
10	1	0	1	0.587	0.580
11	0	−1	−1	0.486	0.480
12	0	1	1	0.545	0.550
13	1	−1	0	0.482	0.490
14	−1	1	0	0.472	0.460
15	−1	−1	0	0.468	0.460
16	0	0	0	0.596	0.610
17	0	−1	1	0.538	0.540

**Table 3:** ANOVA for the response surface quadratic model.

Source	Sum of Squares	Df	Mean Square	*F*-Value	*p*-Value
Model	0.049	9	5.42 × 10^−3^	33.97	< 0.0001
X_1_-liquid–solid ratio	5.67 × 10^−3^	1	5.67 × 10^−3^	35.57	0.0006
X_2_-time	9.90 × 10^−4^	1	9.90 × 10^−4^	6.21	0.0415
X_3_-temperature	5.31 × 10^−3^	1	5.31 × 10^−3^	33.27	0.0007
X_1_X_2_	6.25 × 10^−4^	1	6.25 × 10^−4^	3.92	0.0882
X_1_X_3_	1.32 × 10^−4^	1	1.32 × 10^−4^	0.83	0.3927
X_2_X_3_	7.23 × 10^−5^	1	7.23 × 10^−5^	0.45	0.5224
X_1_^2^	0.013	1	0.013	79.60	< 0.0001
X_2_^2^	0.017	1	0.017	109.53	< 0.0001
X_3_^2^	2.56 × 10^−3^	1	2.56 × 10^−3^	16.05	0.0052
Residual	1.12 × 10^−3^	7	1.59 × 10^−4^		
Lack of Fit	6.25 × 10^−4^	3	2.08 × 10^−4^	1.70	0.3041
Pure Error	4.91 × 10^−4^	4	1.23 × 10^−4^		
Cor Total	0.05	16			
R^2^ = 0.9776; Adj R^2^ = 0.9488; C.V. (%) = 2.33; Adeq precision = 15.210					

**Table 4:** Compounds detected of RP from the peel of *Z. bungeanum* (PZB) by UPLC-MS in positive and negative modes.

Peak No.	t_R_ (min)	[M + H]^+^ (*m*/*z*)	[M − H]^−^ (*m*/*z*)	(ESI^+^) Fragments (*m*/*z*)	(ESI^−^) Fragments (*m*/*z*)	Identification	Type of Compounds
1	3.15	351.27		246, 170, 144		Non-detected	Non-detected
2	3.92		353.36		191, 179,173, 161, 135	Neochlorogenic acid	Phenylpropanoid
3	7.00	355.17		175, 163		Chlorogenic acid	Phenylpropanoid
4	9.12	291.16		273, 165		Epicatechin	Flavonoid
5	10.02		337.24		191, 163	*P*-coumaroylquinic acid	Phenylpropanoid
6	10.67		321.30		244, 173, 129	Non-detected	Non-detected
7	10.88	449.14		287		Cyanidin-3-glucoside	Anthocyanin
8	11.30	595.28		449, 287		Cyanidin-3-rutinoside	Anthocyanin
9	12.55	773.33		303		Quercitrin triglycoside	Flavonoid
10	12.74	627.25		303		Quercitrin bioside	Flavonoid
11	14.14	294.26		276, 205, 177, 135, 121		Non-detected	Non-detected
12	14.42	296.29		278, 207, 120		Non-detected	Non-detected
13	14.90		609.34		300	Rutin	Flavonoid
14	16.31		463.28		300	Hyperoside	Flavonoid
15	16.72		463.33		300	Quercetin-7-glucoside.	Flavonoid
16	17.42		296.34		240	Non-detected	Non-detected
17	17.92		433.25		300	Guaijaverin	Flavonoid
18	19.54		447.29		301, 271, 255, 179, 151	Quercetin-3-rhamnoside	Flavonoid
19	21.87	312.34			262, 145, 105	Non-detected	Non-detected
20	23.09	565.23		302		Non-detected	Non-detected
21	24.32	595.27		287		Kaempferol-3-rutinoside	Flavonoid

**Table 5:** Independent variables and levels of response surface methodology (RSM) experiments.

Independent Variables	Code Levels		
	−1	0	+1
Liquid–solid ratio (X_1_) (mL/g)	25	30	35
Extraction time (X_2_) (min)	30	40	50
Extraction temperature (X_3_) (°C)	20	25	30
